# Protective effects of dietary Kefir against aflatoxin B1‐induced hepatotoxicity in Nile tilapia fish, *Oreochromis niloticus*

**DOI:** 10.1002/fsn3.2838

**Published:** 2022-03-29

**Authors:** Fadia Ben Taheur, Chalbia Mansour, Sondes Mechri, Houcine Laaouar, Sihem Safta Skhiri, Mohamed Bouricha, Bassem Jaouadi, Ridha Mzoughi, Nacim Zouari

**Affiliations:** ^1^ Laboratory of Analysis, Treatment and Valorization of Environmental Pollutants and Products Faculty of Pharmacy University of Monastir Monastir Tunisia; ^2^ Higher Institute of Applied Biology of Medenine (ISBAM) University of Gabes Medenine Tunisia; ^3^ Laboratory of Microbial Biotechnology, Enzymatic and Biomolecules Centre of Biotechnology of Sfax University of Sfax Sfax Tunisia; ^4^ Technical Center of Aquaculture Tunis Tunisia; ^5^ ABCDF Laboratory Faculty of Dental Medicine University of Monastir Monastir Tunisia; ^6^ Ministry of Agriculture, Hydraulic Resources and Fisheries. Agricultural Development Commissionership Gabes Tunisia

**Keywords:** aflatoxin B1, antioxidant stress, histological analysis, probiotic, tilapia, weight gain

## Abstract

The effect of dietary Kefir supplementation on the biometric, biochemical, and histological parameters of Nile tilapia (*Oreochromis niloticus*) exposed to aflatoxin B1 (AFB1, 200 µg/kg diet) contamination was studied. The yeasts were dominant in Kefir followed by lactic and acetic acid bacteria. The Kefir showed relatively interesting antioxidant potential in the DPPH• (IC_50_ = 0.9 ± 0.02 mg/ml) and ABTS•+ (IC_50_ = 2.2 ± 0.03 mg/ml) scavenging activities, Fe^3+^‐reducing power (EC_0.5_ = 1.2 ± 0.01 mg/ml), and β‐carotene bleaching assay (IC_50_ = 3.3 ± 0.02 mg/ml). Three hundred and sixty Nile tilapia weighing 23 ± 5 g were divided into four groups (30 fish/group with 3 replicates), and fed with diets containing Kefir (D2), AFB1 (D3), and Kefir+AFB1 (D4) for 4 weeks, whereas D1 was kept as control group where fish were fed with basal diet. The Kefir supplementation in D4 group significantly increased (*p* < .05) the percent weight gain as compared to D3 group. Moreover, Kefir improved the antioxidant enzymes in the liver, such as catalase (CAT), glutathione peroxidase (GPx), and superoxide dismutase (SOD) activities, that significantly increased (*p* < .05) by 2‐, 3‐, and 1.5‐folds, respectively, as compared to D3 group. The Kefir treatment significantly decreased (*p* < .05) the liver malonaldehyde content by ~50% as compared to D3 group. Histopathological analysis revealed the hepatoprotective effects of Kefir by showing normal liver histological architecture in D4 group, as compared to degenerative changes observed in D3 group. These results suggest that Kefir could be considered as a potential probiotic in Nile tilapia feed to mitigate the AFB1 harmful effects.

## Graphical abstract

Kefir showed interesting in vitro antioxidant and DNA‐protective activities. Aflatoxin B1 (AFB1) enhanced the oxidative stress in the liver. Kefir improved the antioxidant defense systems in Nile tilapia subjected to AFB1. Kefir prevented AFB1‐induced hepatotoxicity in Nile tilapia.

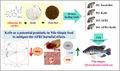

## 1 INTRODUCTION

The Nile tilapia cichlid fish (*Oreochromis niloticus* L.), also called African crucian carp, is a fish cultivated by the freshwater aquaculture industry. It is the second most cultivated species of fish in the world, and its total production has increased by around 10% per year since 2001 and is expected to reach 128 million tons in 2030 (Wei et al., 2021). To cope up with the increasing production of this species, better feed formulations should be provided to meet increasing feed requirements at different growth stages (Xia et al., 2021).

In Tunisia, as a coastal country, fish production is increasing year by year and it reached 1327 tons in 2020. Farmed fish occupied about two thirds of this production, the rest was made up of mollusks and crustaceans. However, fish production has suffered from mycotoxins in the feed during the storage (Ayofemi et al., 2020). The aflatoxins, which were metabolites produced by *Aspergillus* species, such as *A. flavus* and *A. parasiticus*, represented one of the most important mycotoxins types that attack fed fish (Singh & Mehta, 2020).

In recent decades, food contamination with naturally occurring toxic compounds has become a major concern worldwide (Noroozi et al., 2020). Thus, the management of mycotoxin contamination in the fish feed industry was tricky (Yitbarek & Tamir, 2013). In fact, many chemical and physical procedures were used to control mycotoxins in fish feed. However, excessive use of mycotoxin killers could destroy nutrients and lead to a loss of organoleptic quality. The bio‐control of mycotoxins using the catabolic potential of lactic acid bacteria (LAB) and yeasts could be an encouraging way (Mohammadi et al., 2021). The Kefir, as a complex symbiotic association of yeasts and LAB (Ben Taheur et al., 2017), could be a promising avenue for the management of aflatoxin B1 (AFB1) in tilapia fish.

To the best of our knowledge, the effect of Kefir as a dietary supplement in mitigating AFB1 toxicity has not been studied. Thus, the present study aimed to explore some in vitro and in vivo properties of Kefir. The in vitro antioxidant potential was evaluated through various complementary methods, such as Fe^3+^‐reducing power, β‐carotene bleaching assay, radical (DPPH• and ABTS•+)‐scavenging activities, and DNA nicking assay. The protective effects of Kefir administration in tilapia fish on antioxidant enzymes, lipid peroxidation, and hepatic histoarchitecture in AFB1‐induced toxicity was studied.

## 2 MATERIALS AND METHODS

### 2.1 Grain activation and Kefir production

The Kefir grains used in this study were a traditional culture, and the original grain was from the Laboratory of Analysis, Treatment and Valorization of Environmental Pollutants and Products (Faculty of Pharmacy of Monastir, University of Monastir, Monastir, Tunisia). Kefir grains were activated by adding 20 g of grains to 200 ml of commercial ultra‐high‐temperature (UHT) cows' milk, followed by incubation at 25°C. After 24 h, the grains were sieved out and rinsed with sterile distilled water to remove the clotted milk. Then, Kefir grains were placed in 200 ml of fresh UHT milk and incubated under the same conditions. This step was repeated every day for 2 weeks until the grains were used as starter culture in the following experiments (Ben Taheur et al., 2017). The Kefir fermented milk was lyophilized and stored at −20°C until use.

### 2.2 Microbiological analyses

Enumeration and isolation of microorganisms from fermented milk were performed (Ben Taheur et al., 2017). After incubation period of activated grains at 25°C for 24 h in milk, a volume of 10 ml of Kefir was homogenized with 90 ml of physiological sterile water. Serial decimal dilutions were prepared, and 0.1 ml was inoculated in triplicate by surface spreading on specific solid media. LAB were isolated on De Man‐Rogosa‐Sharpe (MRS) agar (Merck) and incubated at 37°C under anaerobic conditions for 3 days. Yeasts were isolated on potato‐dextrose‐agar (PDA) (Merck) at 30°C for 3 days. The acetic bacteria were isolated on M10 agar (composed of 0.5% (w/v) yeast extract, 0.3% (w/v) peptone, 2.5% (w/v) mannitol, and 2% (w/v) agar) and incubated at 30°C for 2 days. All microbial counts were expressed as colony‐forming units per ml of Kefir (CFU/ml).

### 2.3 Antioxidant activities determination

The antioxidant activities of Kefir were assayed using four tests: DPPH• radical‐scavenging activity, β‐carotene bleaching assay, ABTS•+ radical‐scavenging activity, and ferric‐reducing power.

#### 2.3.1 DPPH• radical‐scavenging activity

The DPPH• (2,2‐diphenyl‐1‐picrylhydrazyl) radical‐scavenging activity of Kefir was determined using the method described by Kirby and Schmidt (1997). Briefly, 500 µl of Kefir sample at various concentrations (1–5 mg/ml) was mixed with 375 µl ethanol and 125 µl of DPPH• solution (0.02% (w/v) prepared in ethanol). For each concentration, a blank without DPPH• and a control without sample were prepared. After incubation for 60 min in the dark, the absorbance was measured at 517 nm using UV/VIS T‐80 spectrophotometer (PG Instruments Limited). The IC_50_ value, defined as Kefir concentration needed to scavenge 50% of DPPH•, was determined. BHA (Butylated hydroxyanisole) was used as a positive standard, and the DPPH• reduction was calculated using the Equation (1).

(1) $$\text{DPPH}∙\;\text{radical}‐\text{scavenging}\;\text{activity}\;\left(\%\right)=\left[\left(A_{C}+A_{b}\right)‐A_{S}\right)/A_{C}]\times 100$$

where A_C_, A_b_, and A_S_ represent the absorbances of the control, blank, and sample reaction tubes, respectively.

#### 2.3.2 Ferric‐reducing power

The method described by Yildirim et al. (2001) was used to determine the ferric‐reducing power of Kefir. This assay consisted of estimating the capacity of antioxidant compounds in Kefir to reduce ferric iron (Fe^3+^) to ferrous iron (Fe^2+^). Kefir sample (500 µl) at different concentrations (1–5 mg/ml) was mixed with 1.25 ml 0.2 M sodium phosphate buffer (pH = 6.6) and 1.25 ml 1% (w/v) potassium ferrocyanide (K_3_ Fe(CN)_6_). The mixture was incubated for 30 min at 50°C. Afterward, 1.25 ml 10% (w/v) of trichloroacetic acid (TCA) was added and the mixture was centrifuged at 11,000 × *g* for 10 min. Then, for each concentration sample, 1.25 ml of the resulting supernatant was mixed with 1.25 ml distilled water and 0.25 ml 0.1% (w/v) ferric chloride solution. Absorbance was measured at 700 nm using UV/VIS T‐80 spectrophotometer (PG Instruments Limited). Blanks (without FeCl_3_) were prepared as described previously and the BHA was used as a standard. The reducing power represented the absorbance measured at 700 nm, and the Kefir sample concentration providing 0.5 of the initial absorbance (EC_0.5_) was calculated from the graph of absorbance at 700 nm against sample concentration.

#### 2.3.3 β‐Carotene bleaching assay

The β‐carotene bleaching activity was evaluated using the β‐carotene–linoleic acid assay as described by Koleva et al. (2002). Thus, 0.5 mg of β‐carotene was dissolved in 1 ml of chloroform, and the obtained solution was mixed with 25 μl linoleic acid and 200 μl Tween 40. After chloroform evaporation *in vacuum* at 45°C, 100 ml of bi‐distilled water were added under vigorous agitation. After that, 0.5 ml of Kefir sample (1–5 mg/ml) was mixed with 2.5 ml of β‐carotene‐linoleic acid emulsion and incubated for 2 h at 50°C. The absorbance was measured at 470 nm using UV/VIS T‐80 spectrophotometer (PG Instruments Limited). Control was prepared with the same manner by replacing the Kefir sample by 0.5 ml of distilled water. The IC_50_ value, defined as the Kefir concentration needed to inhibit 50% of β‐carotene peroxidation, was determined. The BHA was used as positive standard. The antioxidant activity was calculated using the Equation (2).

(2) $$\begin{array}{c} \beta ‐\text{carotene}\;\text{bleaching}\;\text{inhibition}\;\text{activity}\;\left(\%\right)\\ =\left[1‐\left(A_{S0}‐A_{\text{S2}})/(A_{C0}‐A_{\text{C2}}\right)\right]\times 100 \end{array}$$

where A_S0_: absorbance of the sample at t = 0, A_S2_: absorbance of the sample at t = 2 h, A_C0_: absorbance of control at t = 0, A_C2_: Absorbance of control at t = 2 h.

#### 2.3.4 ABTS•+ radical‐scavenging activity

The ABTS•+ (2,2’‐azino‐bis‐(3‐ethylbenzothiazoline‐6‐sulphonic acid) radical‐scavenging activity of Kefir was determined according to the colorimetric method described by Re et al. (1999). ABTS•+ stock solution (7 µM) was mixed with 2.45 µM potassium persulfate solution and incubated in the dark at room temperature for 12–16 h. The absorbance of the ABTS•+ solution at 734 nm was adjusted to 0.700 (±0.02) using UV/VIS T‐80 spectrophotometer (PG Instruments Limited) and equilibrated at 30°C. A volume of 3 ml of diluted ABTS•+ solution was added to 30 µl of Kefir sample at different concentrations (1–5 mg/ml) and the absorbance was measured at 734 nm after 6 min (A_t_). The mixture without sample was used as reagent blank reading (A_0_). The IC_50_ value, defined as the Kefir sample concentration needed to scavenge 50% of ABTS•+, was determined. The Trolox was used as a standard. The antioxidant activity of Kefir was expressed in terms of inhibition as given by Equation (3).

(3) $$\text{ABTS}∙+\;\text{radical}‐\text{scavenging}\;\text{activity}\;\left(\%\right)=\left[1‐\left(A/A_{0}\right)\right]\times 100$$

where A: the absorbance of ABTS•+ solution and A_0_: the absorbance of ABTS•+ solution with Kefir sample.

### 2.4 Protective effect on the hydroxyl radical‐induced DNA damage

The DNA nicking assay was performed using pUT57 plasmid. The experiment was performed as described by Jeong et al. (2013) with some modifications (Mechri, Sellem, Bouacem, Jabeur, Chamkha, et al., 2020; Mechri, Sellem, Bouacem, Jabeur, Laribi‐Habchi, et al., 2020). A volume of 5 μl Kefir at different concentrations (12.5, 25 and 50 mg/ml) and 5 μl of plasmid were mixed. Then, 5 μl of Fenton's reagent was added. The mixture was incubated for 30 min at 37°C. The DNA was electrophoresed on 1% agarose gel and visualized.

### 2.5 Experimental diets

The diet (Table 1) was prepared following the method of El‐Sayed et al. (2013) by weighing and mixing the ingredients (fish meal, soybean meal, maize meal, vitamin–mineral mix, and chromic oxide). The soybean oil was added to the dry mix drop by drop with continuous mixing. The Kefir was added by substituting maize meal. The water was added gradually with mixing, until the mixture was molded into small balls. After that, the mixture was divided into four diets. The basal diet D1 was considered as control diet. The D2, D3, and D4 diets were supplemented with Kefir, AFB1, and Kefir+AFB1, respectively, as mentioned in Table 1. The diets were mixed very well to ensure that Kefir and/or AFB1 were evenly distributed throughout the mixture. Then, the diets were ground to a fine powder using a laboratory hammer mill, sieved through a 0.25 mm sieve, and dried at room temperature for 24 h. Finally, the dried diets were stored at −20°C until use.

**TABLE 1:** Formulation and proximate composition of the experimental diets

	Diet			
	D1	D2	D3	D4
Ingredients^a^				
Fish meal	14.00	14.00	14.00	14.00
Soybean meal	45.00	45.00	45.00	45.00
Maize meal	35.00	25.00	35.00	25.00
Soybean oil	4.00	4.00	4.00	4.00
Kefir^b^	0.00	10.00	0.00	10.00
Vitamin‐mineral mix^c^	1.50	1.50	1.50	1.50
Chromic oxide	0.50	0.50	0.50	0.50
AFB1^d^	0.00	0.00	200.00	200.00
Approximate analysis^e^				
Moisture	10.70	18.90	10.68	18.88
Crude protein	28.64	28.84	28.63	28.82
Crude lipid	6.67	6.88	6.65	6.87
Crude fiber	7.06	7.21	7.04	7.20
Ash	6.72	6.51	6.70	6.49
NFE^f^	40.21	31.66	40.30	31.74
Gross energy (kJ/g)^g^	16.02	14.69	16.03	14.69

Note

D1: basal diet; D2, D3, and D4: diets containing Kefir, AFB1, and AFB1+Kefir, respectively.

^a^ g/100 g of diet.

^b^ ml/100 g of diet.

^c^ Vitamin premix contains per kg: Vit. A, 250 000 IU; Vit. D3, 62 500 IU; Vit. K3, 100 mg; Vit. B1, 41 mg; Vit. B2, 150 mg; Vit. B6, 90 mg; Vit. B12, 0.33 mg; Calpan, 175 mg; Folic acid, 20 mg; Biotin, 2 mg; Choline, 2500 IU. Mineral premix contains per kg: Na, 0.35 g; Ca, 0.24 g; Mn, 1.75 g; Fe, 1.5 g; Zn, 1.25 g; Cu, 200 mg; P, 82 mg; I, 10 mg; Co, 8 mg; Se, 7.5 mg.

^d^ μg/kg of diet.

^e^ g/100 g of dry matter.

^f^ Nitrogen‐free extract: 100% – (% lipid + % moisture + % protein + % fiber + % ash).

^g^ Calculated using the factors: carbohydrates, 4.1 kcal 100/g; protein, 5.5 kcal/100 g; lipids, 9.1 kcal/100 g, and transformed to kJ using the factor 4.184.

### 2.6 Fish and experimental protocol

The experimental protocol was conducted at the fish‐culture research station of the National Institute of Marine Sciences and Technologies (Bechima, Gabes, Tunisia). Three hundred and sixty male Nile tilapia (*Oreochromis niloticus* L.), weighing 23 ± 5 g, were randomly maintained in twelve 400 L plastic rearing tanks in batches of 30 fish/tank. The tanks were supplied with water from a geothermal source after undergoing cooling in a large storage tank located upstream of the experimentation system. Three replicate tanks/dietary treatment were used. At the beginning, fish were acclimatized for 15 days prior to the experimental feeding trial, and they were fed a basal diet (D1) daily (Table 1) at a rate of 3% of the body weight/day (Azaza et al., 2008). Then, fish were divided into four groups (30 fish/treatment; three replicates/tank) representing four nutritional groups. The fish of one group remained on the basal diet and were considered as control animals. The other fish groups were switched to the treatment diet containing Kefir (D2), AFB1 (D3), and Kefir+AFB1 (D4). The fish were fed three times daily for 4 weeks. For monitoring growth, the mean body weight/tank was determined weekly by measuring the weight of all the fish in each tank. Water temperature and dissolved oxygen were measured daily using a WTW/Oxi 96 oxythermometer (WTW Measurement Systems Inc.). Levels of pH, nitrite, nitrate, and total ammonium were measured weekly. All measured parameters remained within the tolerance limits known for tilapia. At the end of the experimental period, 30 fish were randomly selected from each treatment (10 from each tank), weighed, and anesthetized with clove essential oil. The livers were removed and weighed. For each fish, a slice of liver was fixed in 10% formaldehyde solution for histological analysis and the rest of the tissues were stored at −20°C for biochemical analyses.

### 2.7 Growth performance parameters

The fish from each tank were weighed at the start and end of the feeding trial to determine initial body weight (IW) and final body weight (FW). The weight gain (WG), percent WG (PWG), feed intake (FI), feed conversion ratio (FCR), hepato‐somatic index (HSI), and survival percentage were calculated using the Equation (4), (5), (6), (7), (8), and (9), respectively.

(4) $$\text{WG}\;\left(g\right)=\left(\text{FW}‐\text{IW}\right)$$

where FW: final body weight and IW: initial body weight.

(5) $$\text{PWG}\;\left(\%\right)=\left[(\text{FW}‐\text{IW})/\text{IW}\right]\times 100$$

where FW: final body weight and IW: initial body weight.

(6) $$\text{FI}\;\left(g/\text{day}/\text{fish}\right)=\text{g dry matter}/\text{day}$$

(7) $$\text{FCR}=\text{dry feed intake}\;\left(g\right)/\text{fish live weight gain}\;\left(g\right)$$

(8) $$\text{HSI}=\left(\text{LW}/\text{FW}\right)$$

where LW: liver weight and FW: final body weight.

(9) $$\text{Survival}\;\left(\%\right)=\left[\text{final surviving fish number}/\text{initial fish number}\right]\times 100$$

### 2.8 Biochemical measurements

All measurements were performed at 4°C to prevent enzyme or tissue degradation. Frozen liver tissues were thawed and homogenized in Tris‐HCl buffer (50 mM Tris‐HCl, 150 mM NaCl, pH = 7.4) in a 1:3 (w/v) ratio using a homogenizer. The homogenates were centrifuged for 25 min at 9,000 × *g* at 4°C, and the resulting supernatants were immediately frozen at −20°C until use. Each biochemical measurement was performed on 20 individual livers from each experimental group and each determination was performed in triplicate.

#### 2.8.1 Total protein content

The total protein content of homogenate samples was measured according to the method of Bradford (Bradford, 1976) using UV/VIS T‐80 spectrophotometer (PG Instruments Limited) at 595 nm. Bovine serum albumin (E^1%^_1 cm_ = 6.7) was used as a standard.

#### 2.8.2 Catalase (CAT) activity

The CAT activity was measured based on the method adapted from (Aebi, 1984). The CAT activity was determined by measuring the decrease of absorbance at 240 nm due to the presence of H_2_O_2_. The reaction mixture contained 990 µl of 0.1 M phosphate buffer (pH = 7.4), 0.1% H_2_O_2_ (30%) and 10 µl of hepatic extract. The optical density was recorded every 15 s for 1 min using UV/VIS T‐80 spectrophotometer (PG Instruments Limited). The CAT activity was expressed in µmol of H_2_O_2_ min^−1^ mg^−1^ protein.

#### 2.8.3 Glutathione peroxidase (GPX) activity

The GPX activity was measured in a coupled enzyme system where NADPH was consumed by glutathione reductase to convert the formed oxidized glutathione form (GSSG) to its reduced form (GSH) according to the method described by Flohé and Günzler (1984) with slight modifications. The decrease of absorbance was monitored at 340 nm using UV/VIS T‐80 spectrophotometer (PG Instruments Limited). The GPX activity was expressed in nmol/min mg^−1^ protein.

#### 2.8.4 Superoxide dismutase (SOD) activity

The SOD activity was determined according to the method described by Sun et al. (1988). The SOD amount capable to inhibit 50% of the reduction rate of nitroblue tetrazolium (NBT) in presence of superoxide anion radicals generated by riboflavin/methionine reaction was measured at 580 nm using UV/VIS T‐80 spectrophotometer (PG Instruments Limited). The enzymatic activity was expressed as unit mg^−1^ protein.

#### 2.8.5 Lipid peroxidation quantification

The lipid peroxidation was estimated from the formation of thiobarbituric acid reactive substance (TBARS) using the method described by Yagi (1976). The TBARS, considered as malonedialdehyde (MDA)‐like peroxide products, were quantified spectrophotometrically by reference to MDA absorbance at 530 nm using UV/VIS T‐80 spectrophotometer (PG Instruments Limited). The lipid peroxidation was expressed in nmol MDA equivalent mg^−1^ protein.

### 2.9 Histological analysis

The livers from control and treated fish were dissected and immediately fixed with 10% formaldehyde solution. After dehydration in a graduated alcohol series, samples were embedded in paraffin. Thin sections of 3 μm were cut, mounted onto glass slides, and subjected to hematoxylin and eosin (H&E) staining for histopathological examination. Preparations were observed and micro‐photographed at 40 × using a Leica Orthoplan microscope (Leica, Solms, Germany) to assess any liver alterations in treated fish as compared to controls.

### 2.10 Statistical analyses

The data were analyzed using a one‐way analysis of variance (ANOVA) to determine differences between experimental groups. Significant differences were determined at the *p* < .05 level using Tukey test. The principal component analysis (PCA) was used to find correlations between the different biomarkers. Statistical analysis was performed using the software Statistica 6.1.478.0 (StatSoft Inc).

## 3 RESULTS AND DISCUSSION

### 3.1 Kefir properties

#### 3.1.1 Microbiological analyses

The total counts of viable microorganisms in Kefir are shown in Table 2. The yeasts were the dominant microbial population followed by LAB and acetic bacteria. These results were consistent with a previous study done on Kefir (Ben Taheur et al., 2017), which reported a high yeasts count as compared to LAB. It was reported that several species of microorganisms were isolated from various Kefir products (Wang et al., 2022). However, the microbial composition of Kefir may differ according to Kefir grains’ origin and the nature of the used milk (Azizi et al., 2021). In the same context, Satir et al. (2015) reported that *Lactobacillus* spp., *Lactococcus* spp., and *Bifidobacterium* spp. counts were higher in Kefir made with goat milk than that made with cow milk. Moreover, Witthuhn et al. (2005) reported the presence of *Lactobacillus plantarum* in the mass cultivated grains, but not in the traditional Kefir grains.

**TABLE 2:** Microorganism counts in Kefir culture after 24 h of fermentation milk

	Lactic acid bacteria	Yeasts	Acetic bacteria
Microorganism counts in Kefir (CFU/ml)	(93 ± 1.41) × 10^6^	(69 ± 2.82) × 10^7^	(45 ± 1.41) × 10^4^

#### 3.1.2 Antioxidant potential

**FIGURE 1:**
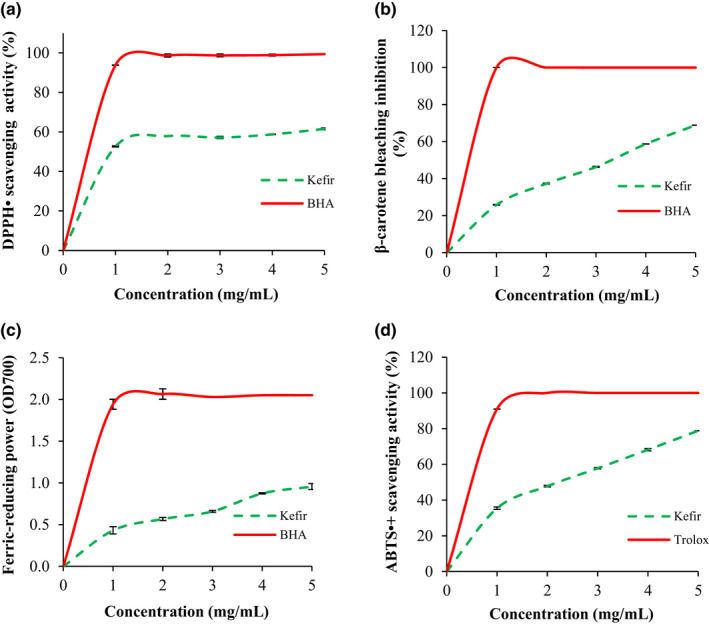
Antioxidant activities of Kefir at different concentrations. (a) DPPH• radical‐scavenging activity; (b) β‐carotene bleaching inhibition; (c) ferric‐reducing power; (d) ABTS•+ radical‐scavenging activity

The antioxidant activities of Kefir were measured using four complementary tests: (i) DPPH• radical‐scavenging activity; (ii) ferric‐reducing power, (iii) β‐carotene bleaching assay, and (iv) ABTS•+ radical‐scavenging activity (Figure 1). Figure 1 shows an increase of the antioxidant activities of Kefir in a dose‐dependent manner. The Kefir showed relatively interesting antioxidant potential in DPPH• scavenging activity (IC_50_ = 0.9 ± 0.02 mg/ml) (Figure 1a), β‐carotene bleaching assay (IC_50_ = 3.3 ± 0.02 mg/ml) (Figure 1b), ferric‐reducing power (EC_0.5_ = 1.2 ± 0.01 mg/ml) (Figure 1c), and ABTS•+ scavenging activity (IC_50_ = 2.2 ± 0.03 mg/ml) (Figure 1d). However, these activities remained lower than the artificial antioxidant BHA. Interestingly, the obtained results were higher than those obtained with peanut‐milk Kefir, where the IC_50_ value for DPPH• radical‐scavenging activity was 5.15 mg/ml and the EC_0.5_ for the ferric‐reducing power was >100 mg/ml (Bensmira & Jiang, 2015). Previous studies reported that goat‐milk Kefir was a promising scavenger for DPPH• radical (Baniasadi et al., 2021) and its antioxidant capacity depended on the fermentation process and storage time (Yilmaz et al., 2016).

**FIGURE 2:**
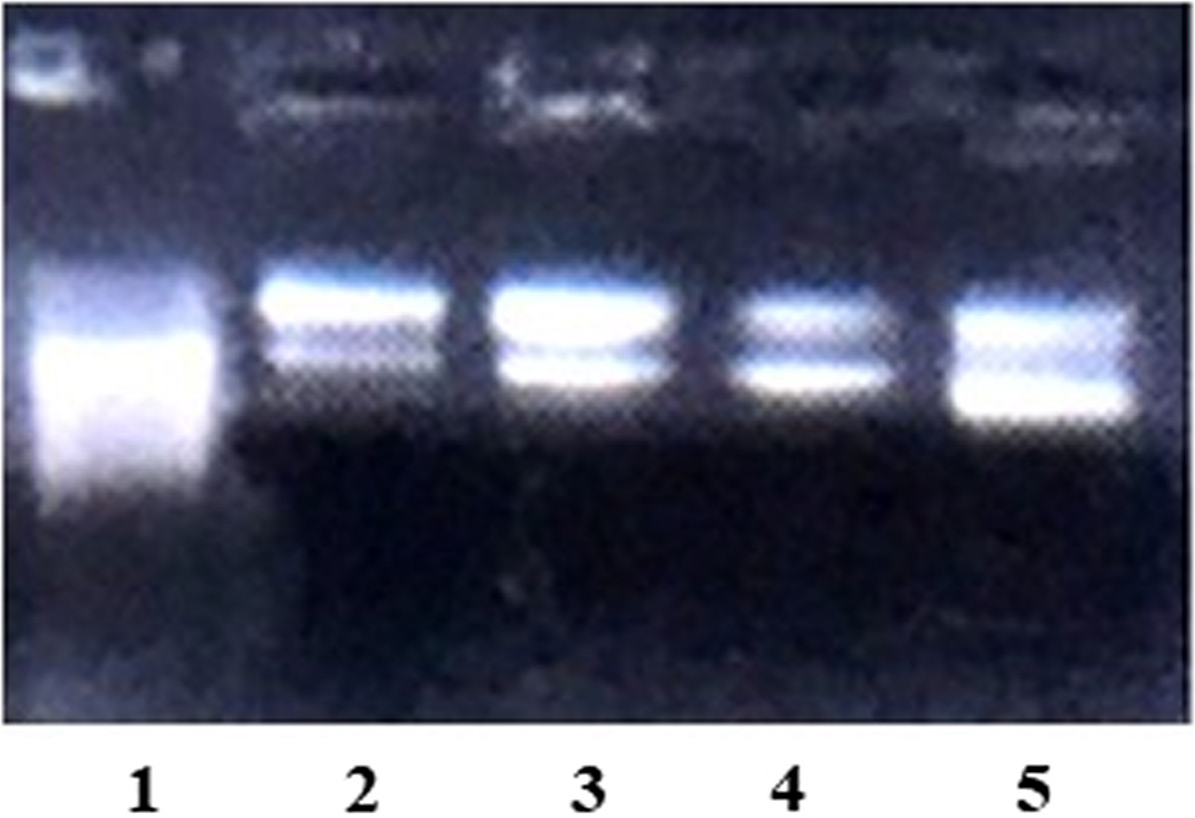
Agarose gel electrophoresis of the OH• radical‐induced DNA scission. Lane 1: pUT57 in the presence of Fenton reagent; lane 2: pUT57 without Fenton reagent; lane 3: pUT57 in the presence of Fenton reagent +50 mg/ml Kefir; lane 4: pUT57 in the presence of Fenton reagent +25 mg/ml Kefir; lane 5: pUT57 in the presence of Fenton reagent +12.5 mg/ml Kefir

The protective effect of Kefir on DNA damage was also studied. Figure 2 shows the electrophoretic pattern of pUT57 plasmid DNA after the Fenton reaction both in the absence and presence of Kefir at different concentrations. In the Fenton reaction, H_2_O_2_ is broken down as a result of electrons transfer from an iron (Fe^2+^) to OH•, which were known to cause oxidative damage in DNA strands. The plasmid without Fenton's reagent was used as a negative control (Lane 2). The migration led to the apparition of three bands: supercoiled, open‐circular, and linear forms. When the plasmid pUT57 was treated with Fenton's reagent (Lane 1), the supercoiled form was completely damaged and converted into the other forms. The OH• radical induced pUT57 plasmid DNA damage and caused the cleavage of supercoiled DNA to the nicked form, indicating a double‐stranded break of pUT57. Interestingly, the addition of Kefir successfully prevented this conversion, which suggest that Kefir protected DNA through its antioxidant activity.

It was reported that the environmental factors, such as oxidative stress, UV light, ionizing radiation, among others can damage DNA, and could contribute to various aging diseases (Mechri, Sellem, Bouacem, Jabeur, Chamkha, et al., 2020; Mechri, Sellem, Bouacem, Jabeur, Laribi‐Habchi, et al., 2020). The Kefir antioxidant potential could be explained by some LAB metabolites that showed chelating activity, reduced metal ions, and protected against oxidation reactions (Wang et al., 2017). In fact, some strains such as *Lactobacillus paracasei* H4‐11, *Lactobacillus fermentum* D1‐1, *Lactobacillus casei* H1‐8, and *Lactobacillus reuteri* H2‐12 showed high antioxidant activity including DPPH• scavenging activity and ferric‐reducing ability (Liu et al., 2020). Additionally, the Kefir antioxidant activities was partly related to the proteolysis of milk proteins by Kefir microflora (Baniasadi et al., 2021). In fact, free amino acids, peptones, and oligopeptides formed following the proteolytic activity of the microorganisms of Kefir contributed to its antioxidant potential (Tagliazucchi et al., 2019). Therefore, Kefir could be used as a natural antioxidant to mitigate the reactive oxygen species production and possibly to prevent tissue damage associated with the oxidative stress.

### 3.2 Growth performance

Four weeks after the experimental trial, no mortality or clinical signs of disease were observed in any group. The PWG, HSI, FI, FCR, and survival percentage were measured (Table 3). No significant difference (*p* > .05) was measured between the different experimental diets for the parameters HSI, FI, FCR, and survival percentage. As compared to the control group (D1), Kefir supplementation (D2) resulted in a slight increase in PWG, whereas AFB1 treatment (D3) significantly decreased (*p* < .05) PWG. The Kefir supplementation in AFB1‐contaminated group (D4) significantly increased (*p* < .05) the PWG as compared to D3 group.

**TABLE 3:** Growth performance of Nile tilapia exposed to four experimental diets: D1: basal diet; D2, D3, and D4: diets containing Kefir, AFB1, and AFB1+Kefir, respectively, for 4 weeks

Parameters	Experimental diets			
	D1	D2	D3	D4
IW (g)	21.62 ± 0.10^a^	21.76 ± 0.31^a^	21.62 ± 0.10^a^	21.60 ± 0.05^a^
FW (g)	44.21 ± 0.41^ab^	45.54 ± 0.87^b^	43.10 ± 1.41^a^	43.88 ± 0.65^ab^
WG (g)	22.98 ± 0.39^ab^	23.79 ± 0.69^b^	21.48 ± 0.36^a^	22.28 ± 0.62^ab^
PWG (%)	106.29 ± 1.79^ab^	109.33 ± 2.86^b^	99.33 ± 2.02^a^	103.15 ± 2.71^ab^
HSI	0.017 ± 0.003^a^	0.019 ± 0.003^a^	0.016 ± 0.004^a^	0.021 ± 0.004^a^
FI (g/day/fish)	1.50 ± 0.01^a^	1.51 ± 0.02^a^	1.47 ± 0.04^a^	1.48 ± 0.02^a^
FCR	1.76 ± 0.02^ab^	1.72 ± 0.03^b^	1.86 ± 0.06^a^	1.79 ± 0.33^ab^
Survival (%)	100±0^a^	100±0^a^	100±0^a^	100±0^a^

Note

Data represent the mean ± S.E. Different letters denote significant differences between treatment groups (*p* < .05).

Abbreviations: FCR, feed conversion ratio; FI, feed intake; FW, final body weight; HSI, hepato‐somatic index;IW, initial body weight; PWG, percent weight gain; WG, weight gain.

Many works reported that several probiotics acted as growth promoters in tilapia aquaculture (Van Hai, 2015). In fact, it was reported that dietary supplementation of probiotics, such as *Psychrobacter maritimus*, *Psychrobacter namhaensis*, *Lactobacillus plantarum*, and *Bacillus paralicheniformis* for 50 days improved growth performance, feed utilization, and immune response of Nile tilapia fingerlings (Hamdan et al., 2016; Makled et al., 2017, 2019; Makled et al., 2020). Furthermore, it was reported that supplementation with *Saccharomyces cerevisiae* for 8 weeks improved the growth performance of Nile tilapia, which was explained by the availability of nutrients due to the immuno‐stimulating compounds in the yeast (Abdel‐Tawwab et al., 2020). An additional study also reported that yeast supplementation for 9 weeks could improve feed digestibility *via* stimulating the digestive enzymes production (Lara‐Flores et al., 2003). However, a reduction in the growth of Nile tilapia exposed to 300 μg AFB1 kg^−1^ for 12 weeks was reported (Hassaan et al., 2020). Deng et al. (2010) showed no effect on the growth of *O. niloticus* and *O. aureus* fed an AFB1‐contaminated diet (245–1641 µg AFB1 kg^−1^) during the first 10 weeks. These authors showed that the administration of this diet for 20 weeks led to a decrease in growth and HSI, as well as abnormal liver morphology (Deng et al., 2010). It seems from the obtained results and those of the literature that the reduction in tilapia body weight depends on the AFB1 dose as well as the exposure period.

### 3.3 Protective effects of Kefir against AFB1‐induced hepatotoxicity

#### 3.3.1 Antioxidant enzymes and lipid peroxidation

The AFB1 was known to be a potent hepatocarcinogen and hepatotoxic mycotoxin (Ben Taheur et al., 2019). In aflatoxicosis, oxidative stress was a common mechanism contributing to the initiation and progression of liver damage. To the best of our knowledge, no research on the effect of Kefir against AFB1‐induced toxicity in tilapia was reported. Thus, the effect of dietary Kefir supplementation, as a probiotic product with antioxidant potential, was studied in tilapia fish contaminated with AFB1.

**FIGURE 3:**
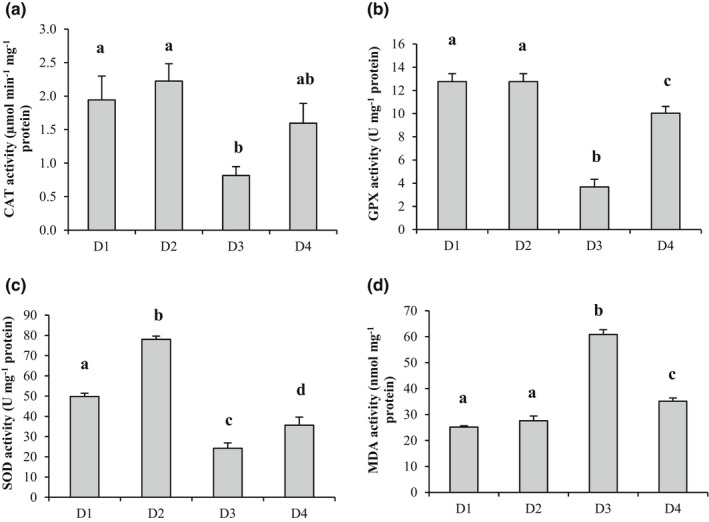
Variations of (a) CAT, (b) GPX, and (c) SOD activities; and (d) MDA content measured in Nile tilapia exposed to four experimental diets (D1‐D4). D1: basal diet; D2: Kefir‐supplemented diet; D3: AFB1‐contaminated diet; and D4: AFB1+Kefir diet. Data represent the mean ± S.E. Different letters denote significant differences between treatment groups (*p* < .05)

Figure 3 shows the effect of different experimental diets on the antioxidant enzymes activities, as well as lipid peroxidation (MDA). As compared to the basal diet (D1), the CAT, GPX, and SOD activities significantly decreased (*p* < .05) in the fish fed on AFB1‐contaminated diet (D3), which was in favor of severe oxidative stress status. Likewise, Hassaan et al. (2020) reported a decrease in antioxidant enzymes CAT and SOD in Nile tilapia following exposure to 300 μg/kg AFB1 for 12 weeks.

de Freitas Souza et al. (2020) also measured a decrease in CAT, GPX, and SOD activities in Nile tilapia after 3 weeks’ exposure to 200 μg/kg AFB1. The CAT, GPX, and SOD were important antioxidant enzymes acting on the reduction of lipid peroxidation and hydrogen peroxide (H_2_O_2_) levels; thus, the reduction of their activities can lead to tissue and cellular damage.

Figure 3d shows the MDA content in all the treated fish, which was used as an indicator of free radical‐induced damage to cell membranes under oxidative stress conditions (Jia et al., 2014). The MDA level was significantly higher (*p* < .05) in fish fed on AFB1‐contaminated diet (D3), which was in accordance with previous studies on Nile tilapia exposed to AFB1. In fact, a relatively higher MDA level was measured in Nile tilapia after exposure to 300 μg/kg AFB1 for 12 weeks (Hassaan et al., 2020) or 200 μg/kg AFB1 for 3 weeks (de Freitas Souza et al., 2020). Interestingly, the Kefir administration to the fish fed on AFB1‐contaminated diet for 4 weeks significantly (*p* < .05) increased the antioxidant enzymes activities and reduced the MDA content, which indicated an improvement of the antioxidant status. As compared to the AFB1‐contaminated diet (D3), the CAT, GPX, and SOD activities increased by 2‐, 3‐, and 1.5‐folds, respectively. Furthermore, the Kefir treatment significantly decreased (*p* < .05) the MDA content by ~50% as compared to D3 group. It is worthy to note that Kefir upregulated antioxidant responses, which could be explained by the potential antioxidant activities measured in Kefir. The antioxidant activities of Kefir could be due to the presence of peptides and probiotics including bacteria and yeasts that were well known by their antioxidant potential. In fact, LAB, such as *Lactobacillus fermentum*, were able to reduce redox damage by a manganese SOD (Songisepp et al., 2004). Additionally, lactobacilli and bifidobacteria Kefir strains could attenuate oxidative status *via* the scavenged free radicals and the decrease of lipid peroxidation (Savini et al., 2010).

On the other hand, vitamins especially vitamins E and C and β‐carotene, as well as exopolysaccharides, mainly Kefiran, produced during milk fermentation showed synergic antioxidant effects (Yilmaz et al., 2018). Furthermore, the reduction of oxidative stress biomarkers could be attributed to the nonavailability of a part of mycotoxins in the fish organisms. Indeed, it was reported that Kefir was able to adsorb AFB1 and consequently reduce their toxicity (Ben Taheur et al., 2017). Previous studies reported a significant correlation between pH reduction and AFB1 breakdown, since the production of organic acids and other fermentation by‐products can detoxify AFB1 (Moradi et al., 2022).

#### 3.3.2 Principal component analysis (PCA)

**FIGURE 4:**
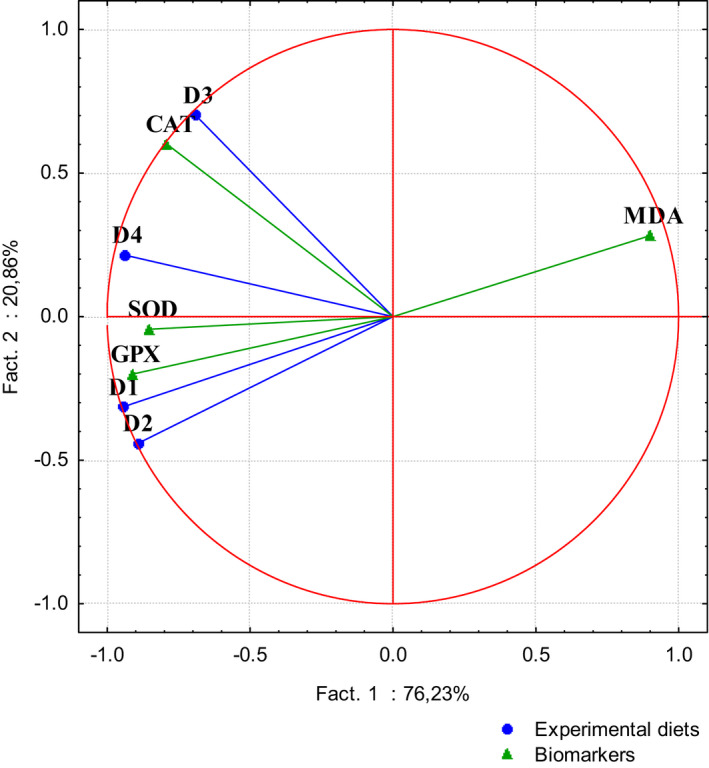
Principal component analysis (PCA) performed on oxidative stress biomarkers measured in liver tissues of Nile tilapia exposed to four experimental diets (D1‐D4). D1: basal diet; D2: Kefir‐supplemented diet; D3: AFB1‐contaminated diet; and D4: AFB1+Kefir diet

The PCA done on measured biomarkers identified two main factors, which explained 97.09% of the total variance (Figure 4). Factor 1 explains 76.23% of the total variance, while factor 2 explained 20.86% of the total variance confirming that D3 group fed on AFB1‐contaminated diet was the most affected. The AFB1+Kefir group (D4) was the least affected as compared to the AFB1 group (D3), which was in accordance with the protective effect of Kefir on the antioxidant status.

The PCA showed a negative correlation between MDA level, and SOD and GPX activities. Pearson's correlation coefficients between the studied biomarkers are presented in Table 4. A correlation coefficient higher than 0.5 was considered as significant at *p* < .05. In the present study, MDA level was negatively correlated with CAT activity (*r* = −.573), GPX activity (*r* = −.836), and SOD activity (*r* = −.677). However, CAT, GPX, and SOD activities were positively correlated.

**TABLE 4:** Pearson's correlation coefficients (*r*) of oxidative stress biomarkers studied in liver tissues in Nile tilapia exposed to the different experimental diets (all experimental groups are included)

	MDA	CAT	SOD	GPX
MDA	1			
CAT	−0.573	1		
SOD	−0.677	0.585	1	
GPX	−0.836	0.616	0.684	1

### 3.4 Histological analysis

**FIGURE 5:**
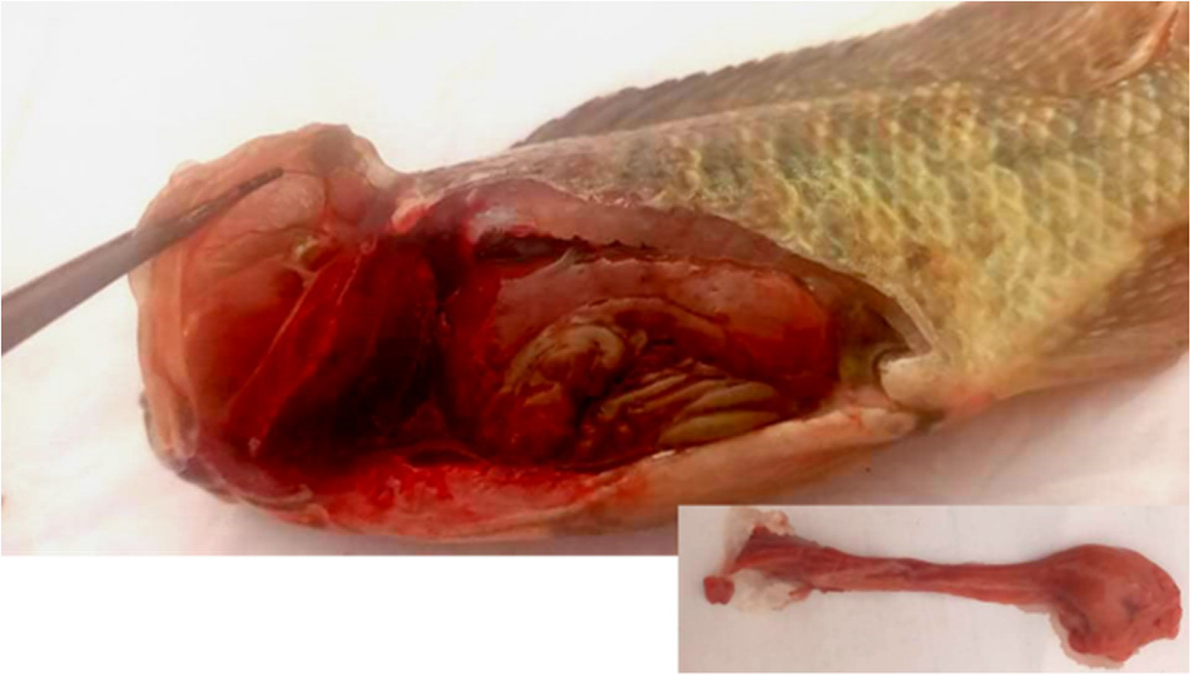
Experimentally AFB1‐contaminated Nile tilapia showing enlarged and pale liver

**FIGURE 6:**
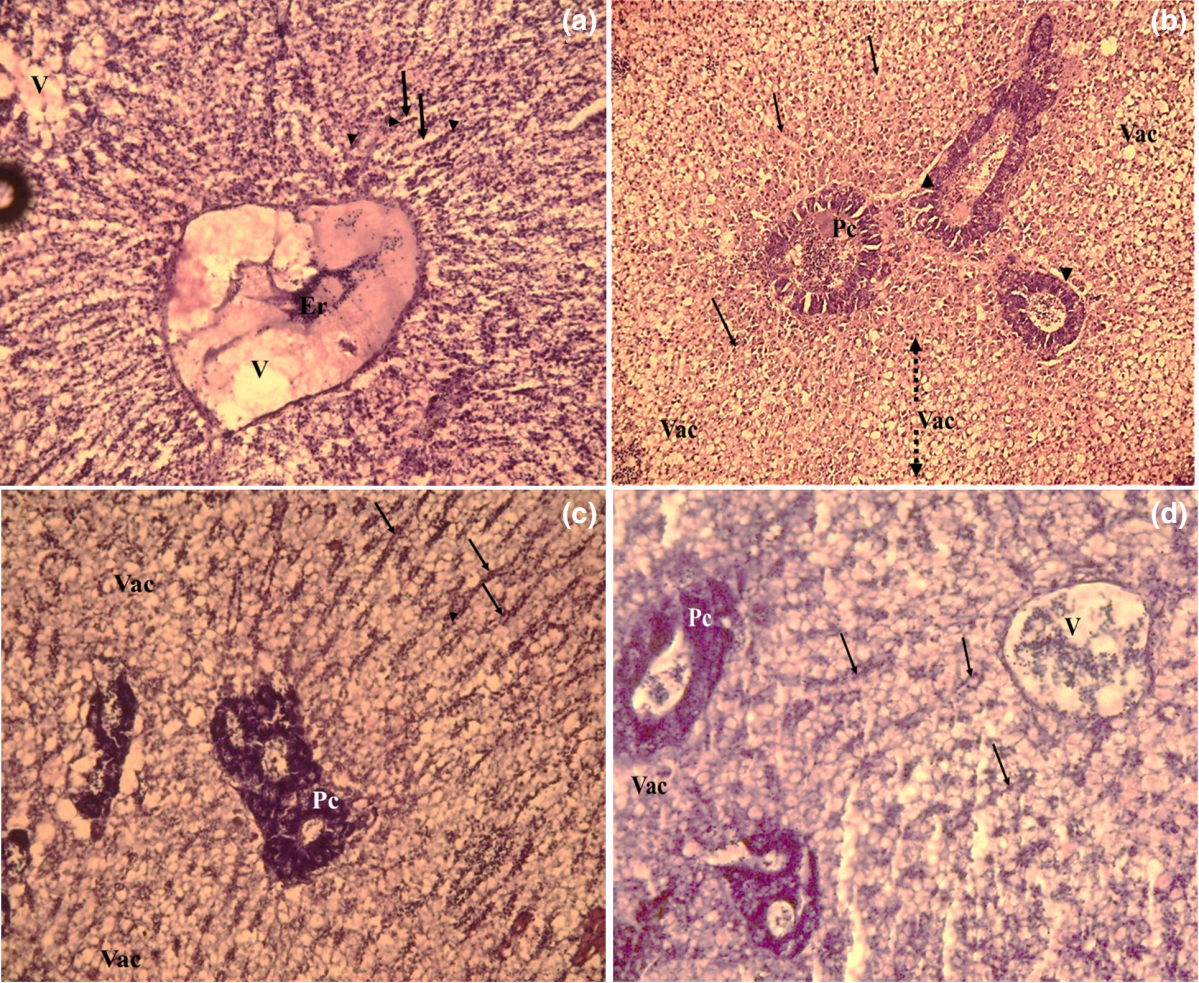
Histopathological sections of H&E liver (×40). (a): control Nile tilapia (D1); (b): Nile tilapia contaminated with AFB1 (D2); (c): Nile tilapia fed on Kefir‐supplemented diet (D3); and (d): Nile tilapia fed on Kefir+AFB1 diet (D4). V, Vein; Er, erythrocytes; Vac, micro‐ and macro‐vacuoles; Pc, pancreas, full arrow: sinusoid vein; discontinuous arrow: lipid vacuoles; head of arrow: hepatocyte nuclei

Post‐*mortem* examination of all studied fish following AFB1 or Kefir treatments did not show abnormal color and appearance of the skin or fins. However, macroscopic examination showed an enlarged and pale liver (Figure 5). The histological study carried out on liver from fish fed on basal diet (D1) or supplemented with Kefir (D2) showed a normal liver structure characterized by normal hepatocytes arranged as cords (Figure 6a,c). Moreover, the liver showed straight sinusoidal veins bounded by hepatocytes, as well as singular micro‐lipid vacuoles. Nevertheless, histology of the liver from tilapia exposed to AFB1‐contaminated diet showed marked changes, such as dilatation, disarrangement, and congestion of the sinusoids, invasion by micro‐ and macro‐lipid vacuoles (lipid accumulation), and impaired contact between the hepatocytes and pancreatic cells (Figure 6b). These histopathological findings were similar to those reported by Abdelhiee et al. (2021). Interestingly, Kefir supplementation in the basal diet improved hepatocytes aspect, and reduced liver damage, lipid vacuoles accumulation, and sinusoid veins disarrangement (Figure 6d).

## 4 CONCLUSIONS

The present study focused on the potentialities of Kefir supplementation in Nile tilapia exposed to aflatoxin B1 contamination, which induced liver damage. Kefir could be suggested as a probiotic food ingredient with interesting antioxidant activities capable of mitigating the harmful effect of AFB1 on Nile tilapia. It is interesting to explore the in silico and molecular exploration studies of Kefir to gain insight into the mechanisms by which the consortium carried out.

## ACKNOWLEDGMENTS

Financial assistance for this study was provided by the Ministry of Higher Education and Scientific Research, Tunisia. Our thanks are due to Dr Houcine Dab (Professor in Animal Physiology at ISBAM) for his fruitful discussion.

## CONFLICT OF INTEREST

The authors declare that they have no conflict of interest.

## Data Availability

All authors confirm that the data supporting the findings of this study are available within the article.
